# Association of tobacco use with periodontal status and salivary biomarkers (thiocyanate and pH) among construction workers: a cross-sectional study

**DOI:** 10.1007/s44445-026-00215-2

**Published:** 2026-07-20

**Authors:** Praneetha Rani, Shivaprasad, Abhijith Shetty, Shilpa Levingston

**Affiliations:** 1 A J Institute of Dental Sciences, Kuntikana, Mangalore, India; 2 Department of Periodontology, Manipal College of Dental Sciences, Manipal Academy of Higher Education, Manipal, India; 3 Peters Dental Clinic, Fujiarah, United Arab Emirates; 4 Srinivas Institute of Dental Sciences, Mukka, India

**Keywords:** Tobacco, Periodontitis, Biomarkers, Thiocyanates, Workers, Saliva, pH

## Abstract

The study aimed to assess and compare the periodontal status, salivary thiocyanate levels and salivary pH among non-tobacco users, smoked tobacco users and smokeless tobacco users. A cross-sectional investigation was carried out on 75 construction workers from active construction sites in Mangalore, Karnataka, India, aged 20–60 years. Participants were categorized into three groups: individuals with no tobacco habit, those consuming smoked tobacco, and those using smokeless tobacco. The periodontal condition of all subjects was evaluated with the WHO Oral Health Assessment Form (2013). Salivary thiocyanate concentration was estimated with an ultraviolet spectrophotometer set at a wavelength of 447 nm, whereas salivary pH was measured using a digital pH meter. Data analysis was performed using the Statistical Package for Social Sciences (SPSS, version 19.0). To compare variations among the groups, one-way analysis of variance (ANOVA) was applied, followed by Tukey’s post hoc test for pairwise comparisons. Gingival bleeding scores were elevated in smokeless tobacco users (100%) compared to smoked tobacco users (88%) and non-users (48%). Periodontal pockets > 6 mm were found in 84% of smoked tobacco users and 76% of smokeless tobacco users, while non-users had none. Mean salivary thiocyanate levels were significantly higher in smoked tobacco users (11.28 ± 1.23) than in smokeless users (7.67 ± 1.08) and non-users (4.26 ± 1.15) (p < 0.001). Salivary pH was lowest in smokeless tobacco users (6.18 ± 0.49), indicating a more acidic environment compared to smoked tobacco users (6.35 ± 0.54) and non-users (7.76 ± 0.45). Both smoked and smokeless tobacco use were associated with poorer periodontal parameters and altered salivary biomarkers among construction workers. Smoked tobacco users demonstrated higher salivary thiocyanate levels, while smokeless tobacco users exhibited lower salivary pH. These findings suggest that salivary biomarkers may reflect tobacco exposure and warrant further investigation regarding their relationship with periodontal disease severity.

## Introduction

Tobacco use is a leading modifiable risk factor for both systemic and oral diseases and continues to be a major global public health challenge. In India, the dual burden of smoked and smokeless tobacco use has contributed significantly to oral morbidity and mortality, particularly due to its association with oral cancers and periodontal disease (Pullishery 2015; Yuvaraj et al. 2020). Periodontal disease, a chronic inflammatory condition, is strongly influenced by behavioral and environmental risk factors, including poor oral hygiene, stress, and the use of tobacco products (Jain et al. 2023). Tobacco consumption not only accelerates disease progression but also modifies the clinical presentation of periodontal inflammation (Adsul et al. 2011).

Smoked and smokeless forms of tobacco affect the oral environment differently. Smoking has been associated with suppressed gingival bleeding, increased pocket depth, and alveolar bone loss primarily due to nicotine’s vasoconstrictive effects (Al-Bayaty et al. 2013). In contrast, smokeless tobacco products such as gutka and khaini, often placed directly in the buccal vestibule, lead to localized gingival recession, mucosal lesions, and increased plaque retention (Winn 2001; Johnson and Slach 2001). These distinct patterns necessitate biomarker-based studies to better understand the impact of various forms of tobacco on oral health.

Saliva, as a diagnostic medium, offers significant advantages due to its non-invasive, cost-effective, and infection-free nature of collection (Wilhelm et al. 2022). It serves as a mirror of systemic and local health, reflecting changes in response to inflammatory and chemical insults. Two such markers of interest in tobacco-exposed individuals are salivary thiocyanate and salivary pH (Sen et al. 2018).

Salivary thiocyanate is a metabolic by-product of cyanide, commonly found in tobacco smoke. Each cigarette delivers 30–200 µg of hydrocyanic acid, which is metabolized in the liver and excreted as thiocyanate in saliva (Hegde et al. 2016a; Kumar et al. 2021). This metabolite serves as a quantitative biomarker of tobacco exposure, with levels in smokers reaching up to 200 mg/day (Flieger et al. 2019). Smokeless tobacco users may also exhibit elevated thiocyanate levels, although absorption pathways differ (Prakruthi et al. 2018). Increased thiocyanate levels, while protective in low concentrations due to their role in oxidative stress buffering, can also participate in nitrosation reactions and potentially promote carcinogenesis (Grover et al. 2016).

Salivary pH, another critical parameter, reflects the buffering capacity of saliva and plays a vital role in maintaining oral microbial balance. A neutral pH range (6.2–7.6) helps prevent enamel demineralization and inhibits the growth of acidogenic bacteria (Fenoll-Palomares et al. 2004). Tobacco, especially in its smokeless form containing slaked lime, may alter the bicarbonate buffer system, leading to salivary acidification and increased risk for caries and periodontal breakdown (Parmar et al. 2017a). Furthermore, reduced pH has been correlated with age-related salivary gland dysfunction and altered electrolyte composition.

Construction workers in India frequently represent a migrant and socioeconomically disadvantaged population with limited access to preventive healthcare services, including oral healthcare (Adsul et al. 2011; Shah et al. 2018). Occupational stress, irregular work schedules, tobacco dependency, poor nutritional practices, and low awareness regarding oral hygiene collectively increase their susceptibility to periodontal disease (Palmer et al. 2005; Zhang et al. 2019). Moreover, tobacco consumption is commonly adopted within this occupational group as a coping mechanism for physical exhaustion and psychosocial stress. Therefore, evaluation of salivary biomarkers in this high-risk occupational cohort may provide insight into non-invasive approaches for early identification of tobacco-associated oral alterations (Humphrey and Williamson 2001; Pullishery 2015). Construction workers, the population studied here, are an especially vulnerable group due to socioeconomic constraints, lack of access to preventive dental care, and high levels of substance use including tobacco. Their occupational stress, irregular schedules, and minimal health literacy contribute to poor oral hygiene practices and delayed diagnosis of periodontal conditions (Mahalakshmi et al. 2022).

Despite numerous studies linking tobacco use to periodontal disease, few have concurrently assessed the clinical periodontal status and salivary biomarkers specifically thiocyanate and pH levels across different types of tobacco use in occupational cohorts. Addressing this gap, the present study aims to assess and compare periodontal status, salivary thiocyanate levels, and salivary pH among non-tobacco users, smoked tobacco users, and smokeless tobacco users. It was hypothesized that both smoked and smokeless tobacco users would exhibit significantly poorer periodontal parameters, elevated salivary thiocyanate levels, and reduced salivary pH compared to non-users, with inter-group variations based on the form of tobacco consumed.

## Methodology

### Study design and setting

A cross-sectional study was conducted among construction workers from active construction sites in Mangalore, Karnataka, India, between August 2021 and February 2022 to determine and compare the periodontal status, salivary thiocyanate levels, and salivary pH among non-tobacco users, smoked tobacco users, and smokeless tobacco users. This study was conducted and reported in accordance with the STROBE (Strengthening the Reporting of Observational Studies in Epidemiology) guidelines.

### Study population and sampling

A total of 75 participants were selected using convenience sampling due to logistical feasibility and accessibility from active construction sites within the age group of 20–60 years where group A, B and C had 25 subjects each.

Group A: Non-tobacco users

Group B: Smoked tobacco users

Group C: Smokeless tobacco users

### Sample size determination

Sample size was calculated based on previous studies (Kumar et al. 2021; Hegde et al. 2016b) assuming a mean difference of 3.5 µmol/L in salivary thiocyanate levels, a standard deviation of 2.8, 80% statistical power, and a significance level (α) of 0.05. The minimum required sample size was estimated to be 23 participants per group. To account for potential variability and data loss, 25 participants were included in each group.

### Inclusion and exclusion criteria

Inclusion criteria: Participants aged 20–60 years who provided written informed consent were included in the study. Eligible individuals were categorized into three groups based on their tobacco usage status: smoked tobacco users, defined as individuals with a history of regular use (≥ 2 years) of combustible tobacco products such as cigarettes and bidis; smokeless tobacco users, defined as individuals with regular use (≥ 2 years) of products such as gutka, khaini, and pan masala; and non-users, defined as individuals with no history of tobacco consumption. Most participants were conversant in Kannada or Hindi. Where necessary, assistance from multilingual translators and site supervisors was utilized to ensure accurate communication and informed consent.

Exclusion criteria: Individuals who reported concurrent use of both smoked and smokeless forms of tobacco were excluded from the study to avoid overlapping exposure effects. Participants with a history of alcohol consumption were also excluded to minimize potential confounding influences on periodontal and salivary parameters. Additionally, individuals with known systemic diseases and those who were unwilling or unable to undergo clinical examination were not included in the study.

The study protocol was approved by the Institutional Ethics Committee of A.J. Institute of Medical Sciences and Research Centre (ECR/348/Inst/KA/2013/RR-16). Written informed consent was obtained from all participants. The study adhered to the principles of the Declaration of Helsinki (2013 revision). Participants identified with poor periodontal status or tobacco-related oral findings were informed about their oral condition and provided brief tobacco cessation counselling and referral information for dental evaluation and treatment.

### Clinical examination

Clinical periodontal assessment was performed using the WHO Oral Health Assessment Form (2013). Examinations were conducted by a single calibrated examiner using a Community Periodontal Index (CPI) probe and a plane mouth mirror.

Clinical examinations were carried out at construction sites under natural daylight supplemented with portable LED illumination. Participants were examined in an upright seated position. Standard infection control protocols were followed, including the use of sterilized instruments and disposable gloves. Intra-examiner reliability was assessed using the Kappa statistic (κ > 0.8), indicating strong agreement.

Duration and frequency of tobacco use were recorded to characterize exposure patterns among tobacco users.

Periodontal Parameters Assessed

Gingival bleeding (as per CPI criteria)

Periodontal pocket depth (categorized as 4–5 mm and ≥ 6 mm)

Although the WHO Oral Health Assessment Form (2013) includes assessment of loss of attachment, the present study restricted periodontal evaluation to CPI-based gingival bleeding and periodontal pocket assessment due to field-based examination constraints at active construction sites, limited examination time, and feasibility considerations. The study was designed primarily as a comparative screening-based assessment rather than a comprehensive periodontal diagnostic evaluation.

###  Sample collection

Unstimulated whole saliva was collected following standardized protocols. Participants were instructed to refrain from eating, drinking, or tobacco use for at least one hour prior to collection. Samples were collected between 9:00 AM and 12:00 PM to minimize diurnal variation. Participants rinsed their mouth with distilled water before expectorating approximately 5 mL of saliva into sterile containers.

*Salivary thiocyanate determination*: Stock Solutions: In 100-mL volumetric flasks, the stock solutions were made: 0.200 M Fe (NO_3_)_3_ in 1 M aqueous HNO_3_ and 2.00 × 10^− 4^ M KSCN in water. For the determination of FeSCN^2+^ complex, standard solutions in test tubes were prepared. The concentration was measured at 447 nm wavelength for each solution. The FeSCN^2+^ concentration in each solution was calculated in which Fe^3+^ > SCN forms FeSCN^2+^ complexes.

### Quantitative analysis (For salivary thiocyanate determination)

**Fig. 1:**
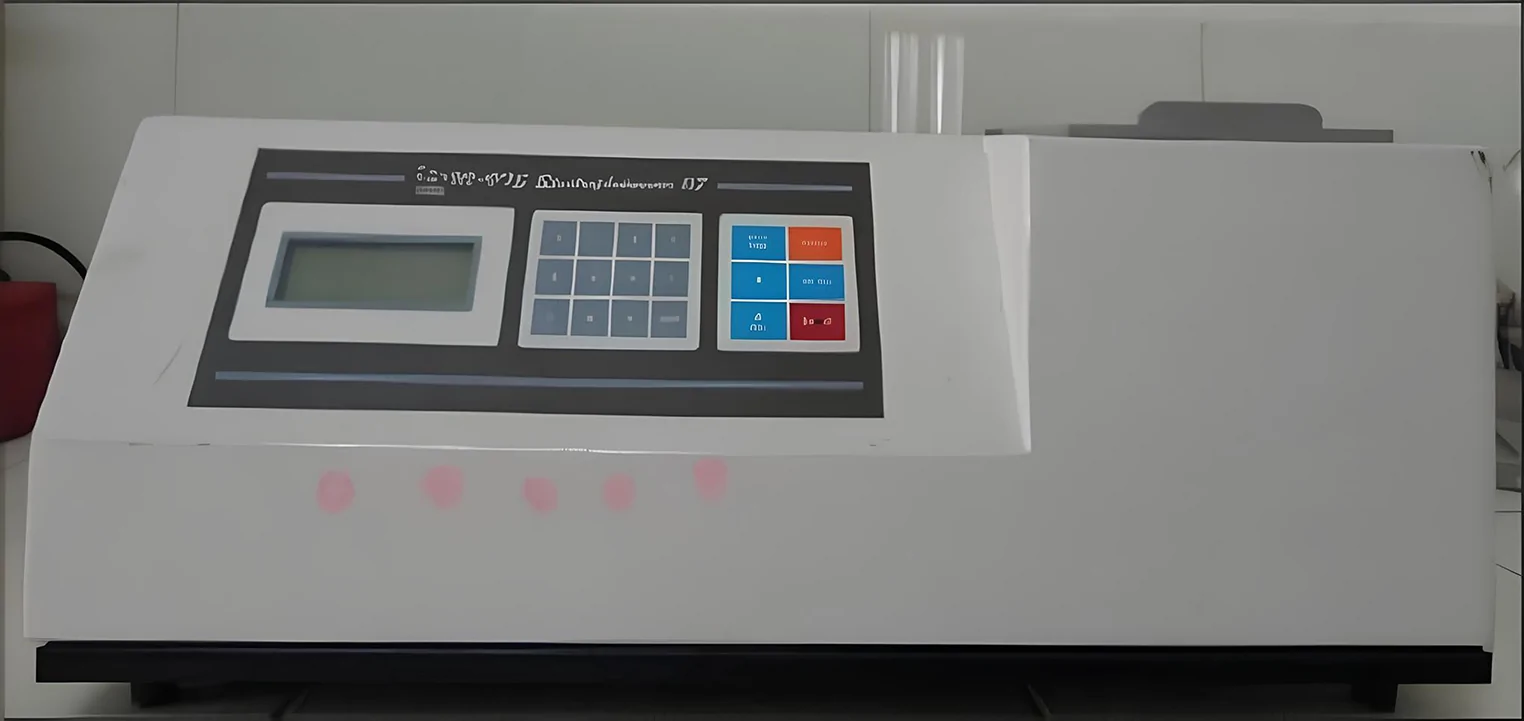
The salivary thiocyanate levels were determined by ultraviolet spectrophotometer

3 ml of saliva was collected in 25 mL beaker. It was transferred to a centrifuge tube and centrifuged at 12,000 rpm for 10 min. After centrifuging 0.5 ml of resulting clear solution was pipetted into 10-mL volumetric flask with Fe (NO_3_)_3_ stock solution. At 447 nm the solution was measured using ultraviolet visible spectrophotometry (Fig. 1) against Fe (NO_3_)_3_ stock solution. Using Lambert and Beer’s law, FeSCN^2+^ concentration in the saliva was determined.

$$\mathrm{Fe}^{3+}+\mathrm{SCN}\qquad \mathrm{FeSCN}^{2+}(\mathrm{Equilibrium})$$

This equilibrium constant was used as a standard in which iron and thiocyanate forms thiocyanate ion. When Fe^3+^ ≥ SCN it doesn’t form thiocyanate complexes since FeSCN^2+^ solutions are deep blood-red in colour, the equilibrium can be measured. The standard curve, from which the concentrations of FeSCN^2+^ are determined, can be measured using standard solutions, in which all the thiocyanate ions are converted to thiocyanate complex ions, FeSCN^2+^.

### Determination of salivary pH

**Fig. 2:**
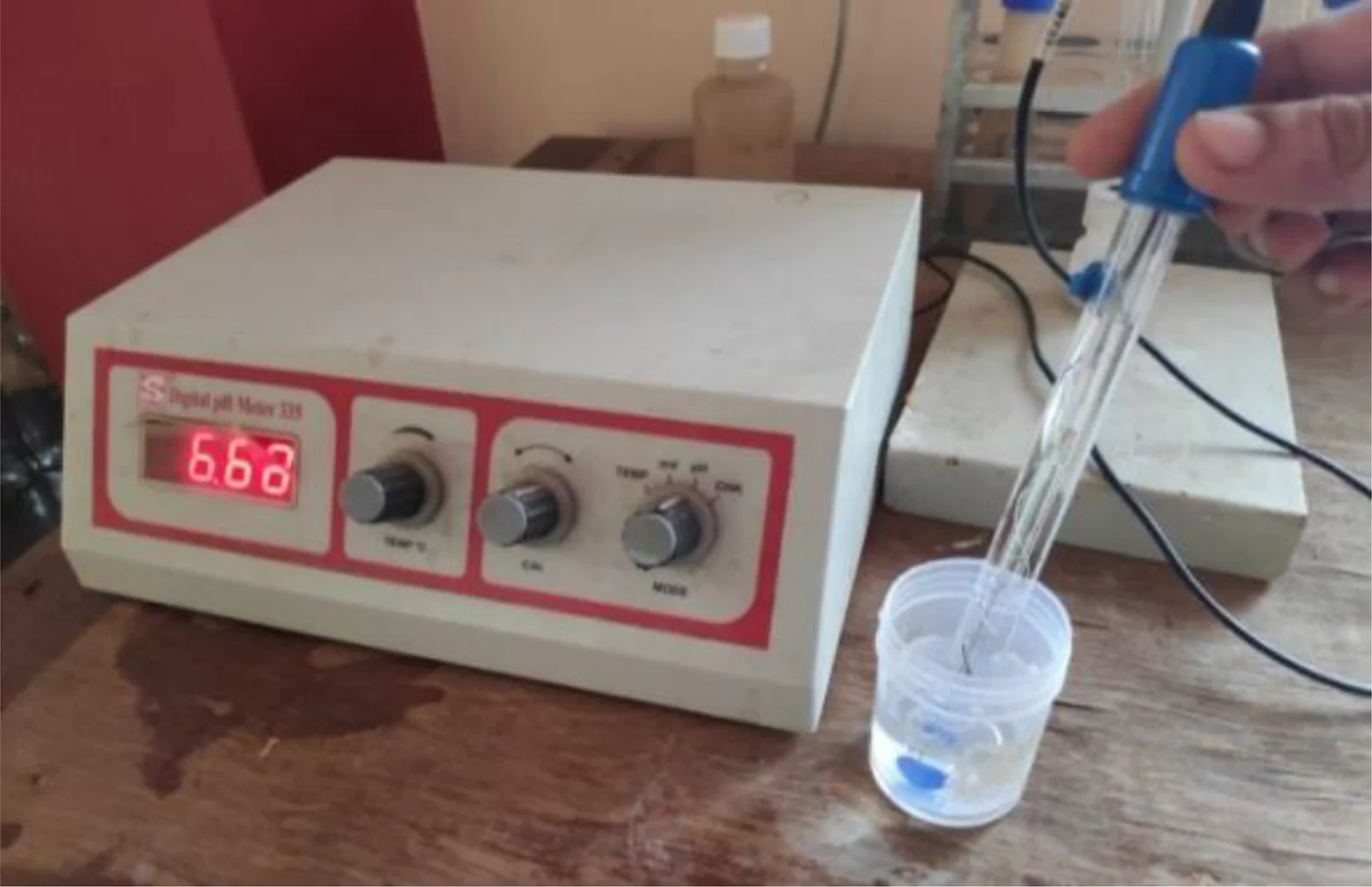
The salivary pH was determined using a pH meter

Salivary pH was measured using a single electrode digital pH meter. The electrode was dipped in 0.1 N hydrochloric acid overnight and was calibrated using freshly prepared buffers of pH 7 and pH 4. It was dried using fresh sterile filter papers each time before using and was dipped in distilled water. After analysing the pH, the electrode tip was again washed with water and then dipped in the double distilled water (Fig. 2).

Confounding Variables: Plaque levels were not recorded in this study, which may act as a potential confounding factor influencing periodontal outcomes.

### Statistical analysis

Statistical analysis was performed using SPSS version 19.0 (IBM Corp., Chicago, IL, USA). Chi-square test was used for categorical variables (gingival bleeding, pocket depth). One-way ANOVA was applied to compare mean salivary thiocyanate and pH levels across groups. Tukey’s post hoc test was used for pairwise comparisons. The level of statistical significance was set at *p* < 0.05.

In cases where ANOVA demonstrated statistical significance but post hoc comparisons were non-significant, findings were interpreted cautiously, considering potential limitations related to sample size and inter-group variability.

## Results

A total of 75 participants were included in the study, equally distributed across the three groups. The mean age of participants was comparable among groups, with no statistically significant differences observed. The majority of participants were male, reflecting the occupational demographics of construction workers. Among tobacco users, both the duration and frequency of tobacco consumption were higher in smoked tobacco users compared to smokeless tobacco users. Oral hygiene practices were suboptimal across all groups, with most participants reporting once-daily toothbrushing and minimal use of adjunctive hygiene aids (Table 1).

**Table 1:** Baseline demographic characteristics, tobacco exposure profile, and oral hygiene practices among study participants

Variable	Non-Tobacco Users (*n* = 25)	Smoked Tobacco Users (*n* = 25)	Smokeless Tobacco Users (*n* = 25)
Age (years, Mean ± SD)	34.2 ± 8.1	36.8 ± 7.5	35.6 ± 8.3
Gender (Male/Female)	20 / 5	23 / 2	22 / 3
Duration of Tobacco Use (years)	—	8.4 ± 3.2	7.1 ± 2.8
Frequency of Use (per day)	—	9.2 ± 3.5 (cigarettes/bidis)	6.8 ± 2.9 (chewing episodes)
**Oral Hygiene Practices**			
Once daily brushing	18 (72%)	20 (80%)	19 (76%)
Twice daily brushing	7 (28%)	5 (20%)	6 (24%)
Use of adjuncts	2 (8%)	1 (4%)	1 (4%)

Although salivary biomarkers and periodontal parameters were comparatively evaluated across tobacco-user groups, participant level biomarker-periodontal correlation analysis could not be performed because only grouped observational data were available for final analysis (Table 2).

**Table 2:** Presents a comprehensive comparison of clinical periodontal parameters and salivary biomarkers among non-tobacco users, smoked tobacco users, and smokeless tobacco users

Parameter	Condition	Non-Tobacco Users *n* (%) or Mean ± SD	Smoked Tobacco Users *n* (%) or Mean ± SD	Smokeless Tobacco Users *n* (%) or Mean ± SD	Statistical Test	Significance (*p*-value)
Gingival Bleeding	Absent	13 (52%)	3 (12%)	0 (0.0%)	Chi-square	*p* < 0.001
	Present	12 (48%)	22 (88%)	25 (100%)		
Periodontal Pocket Depth	Absent	17 (68%)	1 (4%)	0 (0.0%)	Chi-square	*p* < 0.001
	4–5 mm	8 (32%)	3 (12%)	6 (24%)		
	≥ 6 mm	0 (0.0%)	21 (84%)	19 (76%)		
Salivary Thiocyanate	Mean ± SD	4.27 ± 1.15	11.28 ± 1.23	7.67 ± 1.08	ANOVA	*p* < 0.001
	Tukey Post Hoc (vs. Non-Tobacco)	-	*p* = 0.001	*p* = 0.001	Tukey’s Test	Significant
	Tukey Post Hoc (Smoked vs. Smokeless)	-	-	*p* > 0.05		NS
Salivary pH	Mean ± SD	7.76 ± 0.45	6.35 ± 0.54	6.19 ± 0.49	ANOVA	*p* < 0.001
	Tukey Post Hoc (vs. Non-Tobacco)	-	-	*p* = 0.482	Tukey’s Test	Not Significant
	Tukey Post Hoc (Smoked vs. Smokeless)	-	-	NS	NS	NS

ANOVA = Analysis of Variance; NS = Not Significant (*p* > 0.05); SD = Standard Deviation

### Gingival bleeding

The prevalence of gingival bleeding was highest among smokeless tobacco users, with 100% showing signs of bleeding, followed by 88% in smoked tobacco users and only 48% in non-tobacco users. The difference was statistically significant (*p* < 0.001).

### Periodontal pocket depth

Severe periodontal pockets (≥ 6 mm) were most prevalent among smoked tobacco users (84%) and smokeless tobacco users (76%), whereas none were observed in non-tobacco users. Moderate pockets (4–5 mm) were seen more in smokeless tobacco users (24%) compared to smoked tobacco users (12%) and non-tobacco users (32%). A statistically significant difference was observed (*p* < 0.001).

### Salivary thiocyanate levels

The mean salivary thiocyanate levels were highest among smoked tobacco users (11.28 ± 1.23), followed by smokeless tobacco users (7.67 ± 1.08) and non-tobacco users (4.27 ± 1.15). ANOVA analysis revealed a significant difference (*p* < 0.001). Tukey’s post hoc test showed a highly significant difference in thiocyanate levels between both smoked and smokeless tobacco users when compared to non-users (*p* = 0.001). However, the difference between smoked and smokeless users was *p* > 0.05 (not statistically significant).

### Salivary pH levels

The salivary pH was highest in non-tobacco users (7.76 ± 0.45), while smoked (6.35 ± 0.54) and smokeless tobacco users (6.19 ± 0.49) had significantly lower pH levels. Although ANOVA showed a significant overall difference (*p* < 0.001), Tukey’s post hoc test revealed that the difference between the tobacco groups and non-tobacco users was not statistically significant (*p* = 0.482). No significant difference was noted between the two tobacco user groups either.

Although one-way ANOVA demonstrated a statistically significant overall inter-group difference in salivary pH, Tukey-adjusted pairwise comparisons did not reach statistical significance, likely due to conservative correction for multiple comparisons and inter-group variability.

Smoked tobacco users exhibited greater mean duration and frequency of tobacco consumption compared with smokeless tobacco users, which may partly explain the higher salivary thiocyanate levels and increased prevalence of severe periodontal pockets observed in this group. However, formal exposure response correlation analysis was not feasible due to the absence of participant-level analytical modelling.

Overall, these findings underscore the deleterious effects of both smoked and smokeless tobacco use on periodontal health and salivary biomarkers, with smoked tobacco showing particularly elevated thiocyanate levels and deeper periodontal pockets.

## Discussion

The present findings demonstrated significantly poorer periodontal parameters among tobacco users compared with non-users, consistent with previous evidence linking tobacco exposure with periodontal tissue destruction.

To assess oral health, the Community Periodontal Index (CPI) probe was used to measure probing depth, and bleeding upon probing (Ko et al. 2021). Additionally, mouth mirrors provided illumination, indirect vision, and helped retract soft tissues such as the tongue, lips, and buccal mucosa (Bishayi et al. 2023). Spectrophotometric estimation of salivary thiocyanate was measured via UV-visible spectrophotometry at specific wavelengths, and a pH meter was used to determine saliva acidity, as low pH levels can lead to oral health issues like cavities and enamel erosion (Malik 2021).

Although previous literature and research (Al-Bayaty et al. 2013; Baharuddin and Al-Bayaty 2008) suggests that nicotine-mediated vasoconstriction may suppress gingival bleeding among smokers, the present study demonstrated a high prevalence of gingival bleeding among both smoked (88%) and smokeless tobacco users (100%). This discrepancy may reflect poor oral hygiene practices, chronic inflammatory burden, prolonged tobacco exposure, and occupational risk factors within the study population, (Genco et al. 1999; Van Der Weijden and Slot 2011) which may have outweighed the masking vascular effects traditionally attributed to nicotine (Silva 2021).

In the current study, 84% of smoked tobacco users and 76% of smokeless tobacco users had periodontal pockets measuring more than 6 millimetres. The increased periodontal pocket depth observed among tobacco users may be attributed to multiple biological mechanisms associated with tobacco exposure. Nicotine and other tobacco constituents impair neutrophil function, suppress host immune responses, alter cytokine profiles, reduce fibroblast activity, and compromise vascularity within periodontal tissues. Tobacco exposure additionally promotes colonization by anaerobic periodontal pathogens and enhances connective tissue breakdown through increased oxidative stress and matrix metalloproteinase activity, collectively accelerating periodontal destruction. The study findings are concurrent with Bansal et al., since as the illness progresses, periodontal disease increases and becomes more severe over time (Bansal et al. 2015). According to research by Yixin Zhang, use of tobacco increases the inflammatory process associated with periodontal disease by allowing harmful bacteria to invade the gums (Zhang et al. 2019). The factors that influence periodontal disease include an individual’s oral hygiene practices, the existence or absence of bad oral habits, and awareness of dental ailments and diseases in a study by Shah. S (Shah et al. 2018).

In the current study the mean salivary thiocyanate levels of 11.28 ± 1.23 was high in smoked tobacco users when compared to smokeless users. These results are concurrent with Anshul Aggarwal as when smoke is inhaled, it gets absorbed in the lungs, where it is subsequently metabolized into salivary thiocyanate, a byproduct of hydrogen peroxide metabolism, which serves as a useful biomarker for detecting smoked tobacco use in comparison to smokeless tobacco users (Kumar et al. 2021). In another study by Seema Madbhavi smoking more cigarettes over longer periods of time increased the body’s exposure to thiocyanate (Madbhavi and Kale 2019). These results are concurrent with Veena. C (Kalburgi et al. 2014).

In the present study the mean salivary pH of saliva was low smokeless tobacco when compared to smoked tobacco users. These outcomes are concurrent with the results of study by Preetika Parmar, because the usage of lime breaks down bicarbonate, which raises salivary acidity by reacting with the buffering properties of bicarbonate (Palmer et al. 2005). Additionally, saliva buffering systems change saliva’s pH by interacting with ions and electrolytes (Nagarajappa 2013). These results are consistent with Rooban T’s findings (Rooban et al. 2006). According to research by Fenoll-Palomares et al., salivary pH also drops with age since there is a negative correlation between the two (Fenoll-Palomares et al. 2004).

The present study demonstrated concurrent alterations in periodontal parameters and salivary biomarkers among tobacco users. However, direct correlation between biomarker levels and periodontal disease severity could not be established because individual-level analytical modeling was not performed. Future studies incorporating participant-level biomarker-periodontal correlation analysis are necessary to determine the diagnostic relevance of salivary thiocyanate and pH in tobacco-associated periodontal disease.

Inter-group comparison between smoked and smokeless tobacco users demonstrated distinct patterns of periodontal and salivary alterations. Gingival bleeding was more prevalent among smokeless tobacco users, possibly due to direct mucosal irritation, chronic local inflammation, and mechanical trauma at tobacco placement sites (Johnson and Slach 2001; Critchley 2003). In contrast, salivary thiocyanate levels were numerically higher among smoked tobacco users, likely reflecting systemic absorption of combustion-derived cyanide metabolites through pulmonary pathways (Flieger et al. 2019). However, the absence of statistical significance between tobacco-user groups suggests that both forms of tobacco exposure may substantially influence salivary thiocyanate concentrations. Salivary pH was lower among smokeless tobacco users, potentially related to alteration of salivary buffering mechanisms and chemical interactions associated with slaked lime and alkaline additives commonly present in smokeless tobacco preparations (Parmar et al. 2017b).

Smoked tobacco users exhibited greater mean duration and frequency of tobacco consumption compared with smokeless tobacco users, which may partly explain the higher salivary thiocyanate levels and increased prevalence of severe periodontal pockets observed in this group. However, formal exposure-response correlation analysis was not feasible due to the absence of participant-level analytical modeling.

## Limitations

This study has several limitations. The relatively small convenience sample from a single geographic region may limit the generalizability of the findings, and the cross-sectional design precludes causal inference. Clinical attachment loss (CAL/LOA), an important indicator of cumulative periodontal destruction, could not be assessed due to the field-based nature of data collection, limited clinical facilities, and time constraints at active construction sites. Consequently, periodontal status was evaluated using CPI-based screening parameters, which may have underestimated disease severity. In addition, plaque index and other oral hygiene parameters were not recorded and may have acted as confounding factors. Tobacco exposure was self-reported and therefore subject to recall bias. Furthermore, participant-level correlation analyses between salivary biomarkers and periodontal parameters could not be performed. Future studies should incorporate comprehensive periodontal assessment, including CAL, plaque indices, and longitudinal follow-up to validate these findings.

## Implications for clinical practice

The findings of the present study highlight the potential utility of salivary biomarkers, particularly thiocyanate and pH, as non-invasive adjunctive tools for identifying individuals at increased risk of tobacco-associated periodontal alterations. In resource-limited and community-based settings, such as construction sites, saliva-based screening may facilitate early detection and prompt referral for periodontal evaluation. Additionally, the observed differences in periodontal parameters among tobacco users underscore the need for targeted preventive and cessation strategies within high-risk occupational groups.

## Implications for future research

Future studies conducted in occupational settings should consider the use of mobile dental units, portable examination equipment, and dedicated workplace screening programs to facilitate comprehensive periodontal assessment, including clinical attachment loss measurements. Standardized examiner calibration repeated clinical evaluations, and multicentric recruitment strategies may further improve data quality and external validity. Longitudinal cohort studies evaluating exposure-response relationships between tobacco consumption patterns, salivary biomarkers, and periodontal disease progression would provide stronger evidence regarding the diagnostic utility of salivary thiocyanate and pH.

## Conclusion

The present study demonstrated that both smoked and smokeless tobacco use were associated with adverse periodontal findings and altered salivary biomarker profiles among construction workers. Smoked tobacco users exhibited higher salivary thiocyanate levels, whereas smokeless tobacco users demonstrated lower salivary pH. These biomarkers may reflect tobacco exposure; however, further longitudinal and correlation-based studies are required to establish their role in predicting periodontal disease severity.

## Data Availability

No datasets were generated or analysed during the current study.

## Declarations

### Author information

Nil.
